# Lithological Heterogeneity and Its Impact on Soil Settlements at the Building Scale

**DOI:** 10.1007/s10706-025-03157-4

**Published:** 2025-05-29

**Authors:** Alfonso Prosperi, Tom de Gast, Paul A. Korswagen, Mandy Korff, Jan G. Rots

**Affiliations:** 1 Faculty of Civil Engineering and Geosciences, Delft University of Technology, Stevinweg 1, 2628 CN Delft, 2600 GA Delft, The Netherlands; 2 Deltares, P.O BOX, 177, 2600 MH Delft, The Netherlands

**Keywords:** Soil heterogeneity, Settlement, Subsidence, Numerical models, Building damage

## Abstract

Soil heterogeneity, due to variations in the subsurface stratigraphy or properties within a layer, can trigger or amplify differential settlements that affect buildings and infrastructure and can thus lead to (increase in) damage. The state-of-the-art mainly focuses on the effect of heterogeneous properties within a layer on engineering problems. From this, it is known that the variation in properties can increase the vulnerability of a structure. However, nearly always variations in the soil lithological conditions are disregarded, while they can influence subsidence potentially even more. Lithological variations are relevant both at the scale of individual buildings as well as different scales (city, regional, country), for which often detailed soil information is not available. Thus, for a better prediction of potential building damage related to subsidence, knowledge about the scale and influence of lithological variations is needed. This paper describes an approach to quantify and investigate the influence of lithological heterogeneity at the scale of a single building. Moreover, this exploratory study evaluates the influence of lithological heterogeneity on the spatial variability of settlements, intending to upscale the approach to regional application. Two independent datasets at high resolution (site-specific) and low resolution (national level) are used to retrieve the stratigraphic conditions for the area selected for the analyses. One-, Two- and Three-dimensional numerical models, based on the collected information are used to simulate the consolidation process and settlement due to a uniform load imposed on the surface level of the study area. Additional analyses investigate the influence of loading conditions and groundwater table. The parameter “correlation length” is used to quantify the spatial variability of the soil layer thickness and then of the computed settlements. The analyses reveal that the spatial variability of the soil strata thickness matches that of the computed settlements, ranging from 2 to 10 meters. In other words, the lithological variability of the soil leads to differential settlements occurring at the scale of man-made structures such as houses, roads, and embankments. Thus, the results encourage including the contribution of lithological heterogeneity in models and predictions of differential settlement at the scale of individual structures. Moreover, the statistical properties, in terms of mean, spread and distribution shape, of the settlement computed through in-situ specific models, match with those derived at the national scale. These results are expected to support the identification of areas potentially influenced by lithological soil heterogeneity, thus showing potential for upscaling to regional or national levels.

## Introduction

Both natural and man-made soils are strongly characterized by an anisotropic behaviour and spatial heterogeneity that originates from different depositional processes and loading histories (Elkateb et al. 2003; Breysse et al. 2005; Uzielli et al. 2006; Yuan et al. 2024).

Soil heterogeneity has been distinguished (Elkateb et al. 2003; Houy et al. 2005; Popescu et al. 2005; Viviescas et al. 2022) in two types: (i) inherent spatial soil variability, which describes the variability of the soil properties, such as the compressibility or permeability, from one point to another within a layer, and (ii) lithological heterogeneity, which refers to the variation of the stratigraphic information, such as lithology, unit thickness, inclination of the soil strata. Soil heterogeneity affects soil behaviour and can influence different engineering problems (Bauduin 2003; Elkateb et al. 2003; Breysse et al. 2005; Costa et al. 2020; Peduto et al. 2022; Abija 2023; Fiamingo et al. 2023). Subsidence serves as a prime example wherein soil heterogeneity can enhance both the magnitude and the spatial distribution of the ground settlements (Gibson 1974; Breysse et al. 2005; Verberne et al. 2024).

In the Netherlands, many urban areas are exposed to the occurrence of subsidence processes, due to the presence of soft soils. Soft soils are susceptible to settlement because of their low bearing capacity and high deformability (Peduto et al. 2017; Koster et al. 2018; Peduto et al. 2022; Prosperi et al. 2022; Basha et al. 2024; Faleih et al. 2024). The settlement can be further exacerbated by local lithological heterogeneities, e.g., the variable thickness of soft soils beneath the buildings.

In this context, identifying, measuring and quantifying sources of soil heterogeneity represents a key task, as they could lead to uneven settlement and, in turn, damage to buildings. Moreover, it is crucial to identify areas prone to differential settlements due to lithological heterogeneity, as they represent hotspots for which appropriate risk mitigation strategies can be addressed.

In the current state of research, previous studies typically include the variability of soil properties and their influence on soil-structure problems and foundation settlements (Breysse et al. 2005; Houy et al. 2005; Popescu et al. 2005; Kumar et al. 2024; Zhang et al. 2024). The results of these works have shown that variation in soil properties within a layer can augment differential settlements (Breysse et al. 2005; Houy et al. 2005; Jamshidi Chenari et al. 2019). Conversely, only a few analyses focused on the effects of stratigraphic variations, e.g., (Conte et al. 2004; Han et al. 2007; Krzysztof 2019; Ibrahim and Al-Obaydi 2021; Peduto et al. 2022).

This study aims to investigate how soil lithological heterogeneity, including variations in soil types and their thickness, influences the settlements occurring at the scale of existing structures such as houses, roads, and embankments. Toward this aim, this analysis focuses on an area selected for the availability of 100 closely spaced Cone Penetration Tests (CPTs) (de Gast 2020). The available CPTs offer detailed insight into subsurface stratigraphy and the spatial variation of the soil strata. Then, numerical analyses are used to evaluate the soil response and to assess the relationship between lithological heterogeneity and the spatial distribution of the computed settlements. The results are expected to provide knowledge for further studies that focus on how lithological heterogeneity can influence the damage to buildings. For instance, numerical models in which the building is coupled to the subsoil can be used to assess the damage due to differential settlement enhanced by soil lithological heterogeneity.

Additionally, the stratigraphic information of the study area was retrieved from an available 3D “GeoTOP” model (www.dinoloket.nl) that discretizes the subsurface at the national level and is used as input for additional numerical analyses. Hence, the results of the site-specific numerical models are compared to the models based on lower resolution (national level). The goal of this comparison is to assess whether subsurface models with lower resolution than in-situ specific data can still capture the effects of local soil lithological heterogeneity on settlement occurrence.

The paper begins with a description of the methodology in Sect. 2. The results are presented in Sect. 3. In Sect. 4 the findings of this study are discussed, and the main conclusions are presented in Sect. 5.

## Methodology

**Fig. 1:**
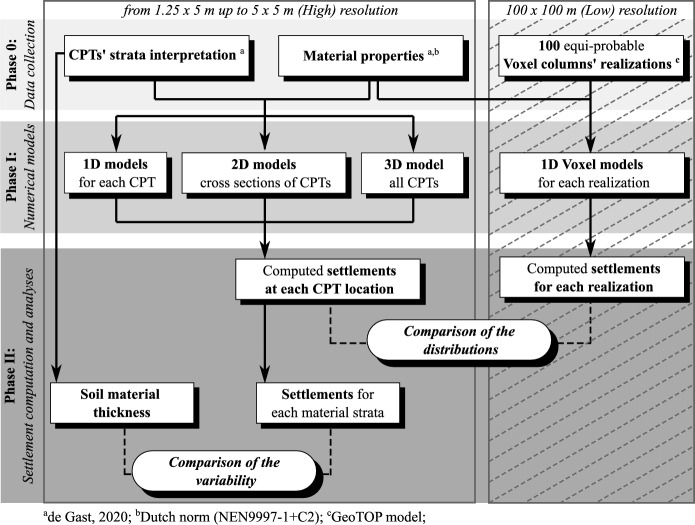
Flowchart of the adopted procedure

The adopted methodological approach consists of three phases (Phase 0, I, and II in Fig. 1):

In Phase 0, data at different resolutions are collected over the study area, including information about the stratigraphy and the hydro-geo-mechanical material properties. The correlation length, which represents the distance over which observations remain significantly correlated, is computed to quantify the variability of the thickness of each soil layer identified in the stratigraphy. Subsoil information, retrieved from the highest resolution dataset (in-situ specific), and available hydro-geo-mechanical properties of the soil materials are then used as the inputs for the numerical models. These numerical analyses are carried out to investigate the influence of soil lithological heterogeneity on the settlement occurrence at the scale of buildings.

Toward this aim, one-, two- and three-dimensional numerical models are built, based on the in-situ specific information. This includes the stratigraphic data and material properties, which are utilized to construct the models. Thus, settlements are computed with the numerical models, further distinguishing the contribution of each soil layer. Additional 1D models, based on the stratigraphic information provided by the national-level subsurface model, are used to evaluate the response of the study area. Sensitivity analyses are further carried out to study the influence of the groundwater table and the different loading conditions on the settlement occurrence and its spatial variability.

In Phase II, the settlements computed using the finite element analyses are retrieved. First, a comparison between the in-situ specific 1D, 2D and 3D numerical models is carried out to investigate the differences. For each soil stratum, the correlation length is computed using the computed settlements and compared with that of the material thickness. This step allows for an investigation into whether the two metrics can be correlated.

Finally, the results of the 1D, 2D and 3D analyses at the scale of the study area are compared with those of the 1D models at the national scale to evaluate the effects of the different resolutions and to determine the effect of lithological variations on the building scale. The comparison aims to assess whether national-resolution models can adequately represent the effects of locally heterogeneous soil stratigraphy. To this end, the distribution of the computed settlements for each analysis is obtained, and the shape, mean, and dispersion of the distributions are compared.

### Case Study and Available Datasets

**Fig. 2:**
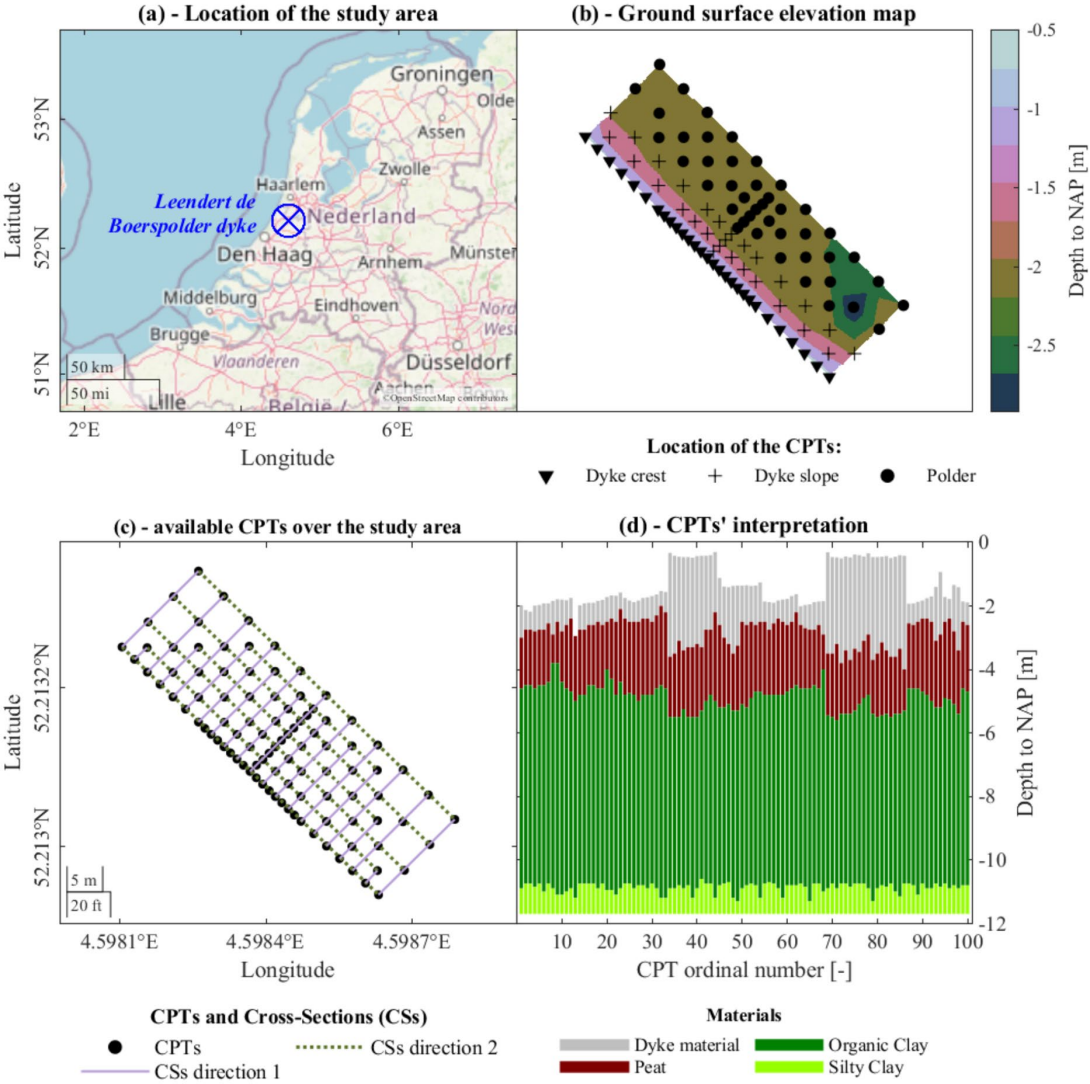
Location of the study area and the available cone penetration tests (CPTs). **a** show the location of the study area, plot **b** shows the elevation maps, **c** shows the selected cross-sections over the study area and **d** shows the stratigraphy at each location based on the interpretation of the CPT data (de Gast 2020)

The area selected for the analysis is the field of the *Leendert de Boerspolder* dyke (Fig. 2a), in the northwest of the Netherlands (de Gast 2020). The available information encompasses: (i) 100 CPTs data (Fig. 2b and c), their location and interpretation in terms of lithotypes up to a depth of 11.70 m to NAP (i.e., the *Amsterdam Ordnance Datum* or *Normaal Amsterdams Peil* in Dutch) (Fig. 2d), (ii) hydro-geo-mechanical material properties (Table 1) (de Gast 2020; de Gast et al. 2021a).

**Table 1:** Material properties of each soil layer adopted in the numerical models

Parameter	Symbol	Unit of measure	Source	Materials			
				Dyke material/sand	Peat	Organic Clay	Silty clay
Dry unit weight	γ_dry_	kN/m^3^	1	13.00	9.00	14.50	14.00
Saturated unit weight	γ_sat_	kN/m^3^	1	18.00	10.00	15.00	17.00
Modified compression index	λ*	–	2	4.99 × 10^−2^	2.00 × 10^−1^	9.99 × 10^−2^	2.22 × 10^−2^
Modified swelling index	κ*	–	2	2.50 × 10^−2^	9.99 × 10^−2^	5.00 × 10^−2^	1.11 × 10^−2^
Modified creep index	μ*	–	2	2.00 × 10^−3^	1.00 × 10^−2^	5.00 × 10^−3^	9.00 × 10^−4^
Effective cohesion	c’	kN/m^2^	1	5.00	2.50	4.40	1.90
Effective friction angle	φ’	°	1	33.00	28.80	29.50	30.00
Pre-overburden pressure	POP	kN/m^2^	3	2.00	22.00	20.00	12.00
Hydraulic conductivity	k	m/day	1	3.46 × 10^−1^	4.00 × 10^−2^	7.52 × 10^−4^	7.52 × 10^−4^

^1^de Gast 2020 (de Gast 2020)

^2^NEN9997−1 + C2 (NEN9997−1 + C2:2017 nl, 2017)

^3^Estimate

The closely spaced CPTs, located along the crest of the dyke and adjacent to the dyke, cover the study area of about 15 × 50 m with an uneven grid (Fig. 2b and c) (de Gast et al. 2017, 2020, 2021b). In particular, 29 CPTs were taken along the crest of the dyke, 28 CPTs along the slope and the remaining in the polder area (Fig. 2b) (De Gast et al. 2021b). The distance between two adjacent CPTs varies from about 1.25 m up to 5.0 m, and the coarsest spacing of the CPTs’ grid is equal to 5 m × 5 m (de Gast et al. 2017, 2020). Thus, this dataset characterizes the study area at the in-situ level, hereinafter referred to as “high” resolution. The in-situ measurements reveal the presence of four soil strata, herein labelled as (de Gast et al. 2017, 2021a; de Gast 2020):

- *dyke material* a mix of clay, silt, sand and rubble that had been placed periodically starting from ~ 1600 AD to form the embankment. The building and maintenance of this man-made layer have caused the underlying layers to compress (de Gast et al. 2017).
- *Peat* organic layer affected by the overlying dyke materials.
- *Organic clay* clay in which the organic content decreases with depth.
- *Silty clay* clay layer rich in silt, that continues until ~ NAP − 16.0 m depth, beyond which sand is found.

The material properties shown in Table 1 are based on laboratory investigations (de Gast 2020; de Gast et al. 2021a), further integrated by values available in the Dutch norm (NEN9997−1 + C2:2017 nl 2017) and estimates (de Gast 2020; de Gast et al. 2021a).

Additional subsoil information is collected from the “GeoTOP model” (www.dinoloket.nl), which discretizes the surface area of the Netherlands (at the national level) up to a depth of 50 m in rectangular blocks, namely “Voxels”, each measuring 100 m × 100 m × 0.5 m (height × width × depth) (Stafleu et al. 2011, 2019; Kruiver et al. 2017). The data used to derive the GeoTOP model do not include the 100 CPTs available in the study area, and thus the two datasets are independent. Due to the size of the Voxels, this set of data is herein referred to, for the study area as “low” resolution, if compared with the closely spaced CPTs. The GeoTOP model provides estimates of the lithological classes for each Voxel (Stafleu et al. 2019): Anthropogenic material, Organic material (Peat), Clay, Clayey sand, silty clay and loam, and Sand, distinguished according to the grain size in “Fine Sand”, “Medium Sand” and “Coarse sand” and “Gravel”. The lithological classes assigned to each Voxel are the outcome of interpolation techniques. The procedure, carried out in previous studies, results in a set of 100 statistically equally likely realizations for each Voxel. For additional details, the reader is referred to (Stafleu et al. 2019).

In other words, 100 realizations, obtained from prior research, provide estimates of the soil type that lies within each Voxel, i.e., a cell that discretizes the surface at a certain depth. The group of Voxels that discretizes the subsurface along the depth, i.e., Voxels that are piled on top of each other, at a certain location is herein referred to as the “Voxel column”.

**Fig. 3:**
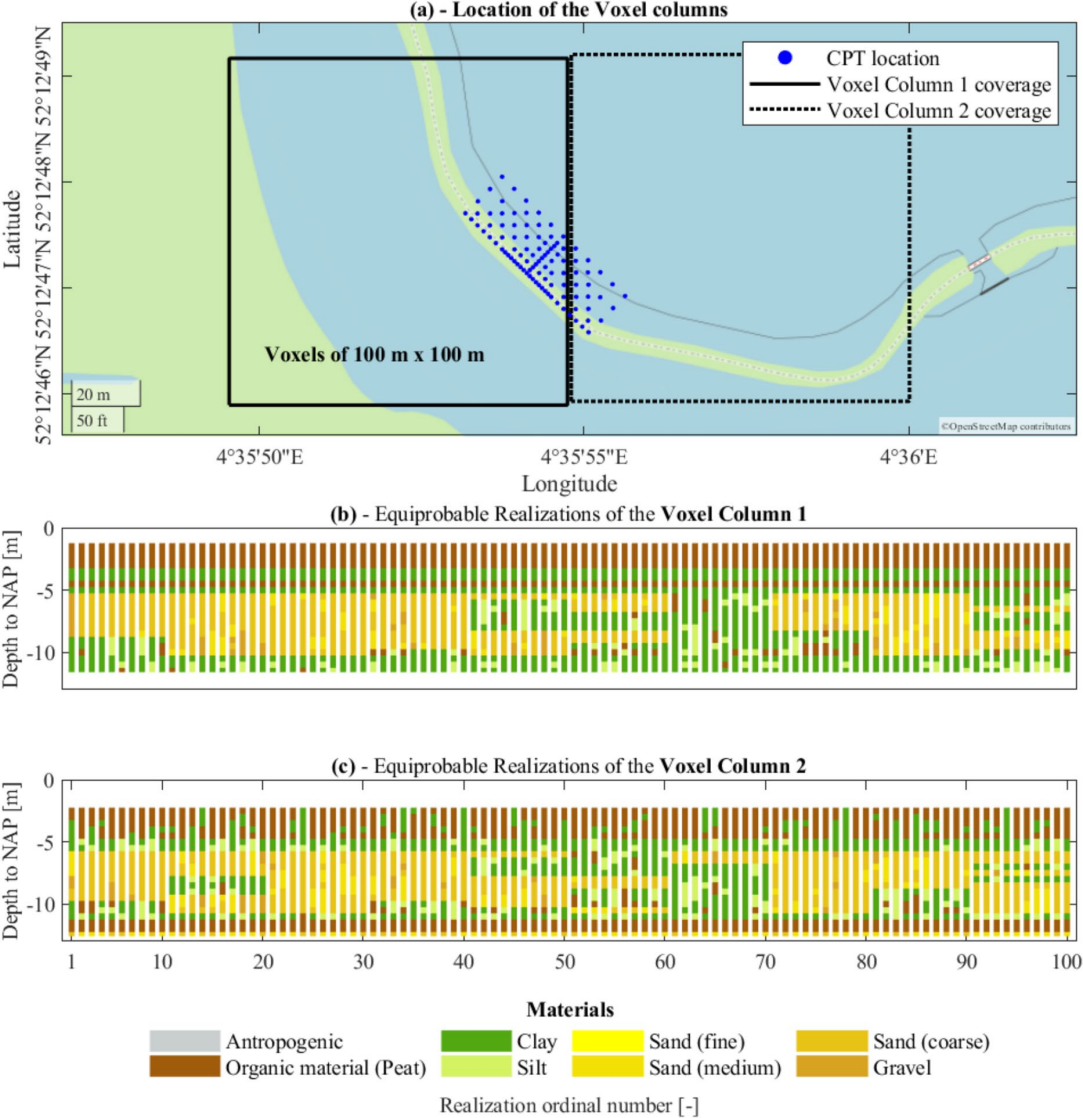
The Voxel columns from the available GeoTOP model: **a** the location of the Voxel columns, **b** and **c** the 100 equiprobable realizations for columns 1 and 2 respectively

**Fig. 4:**
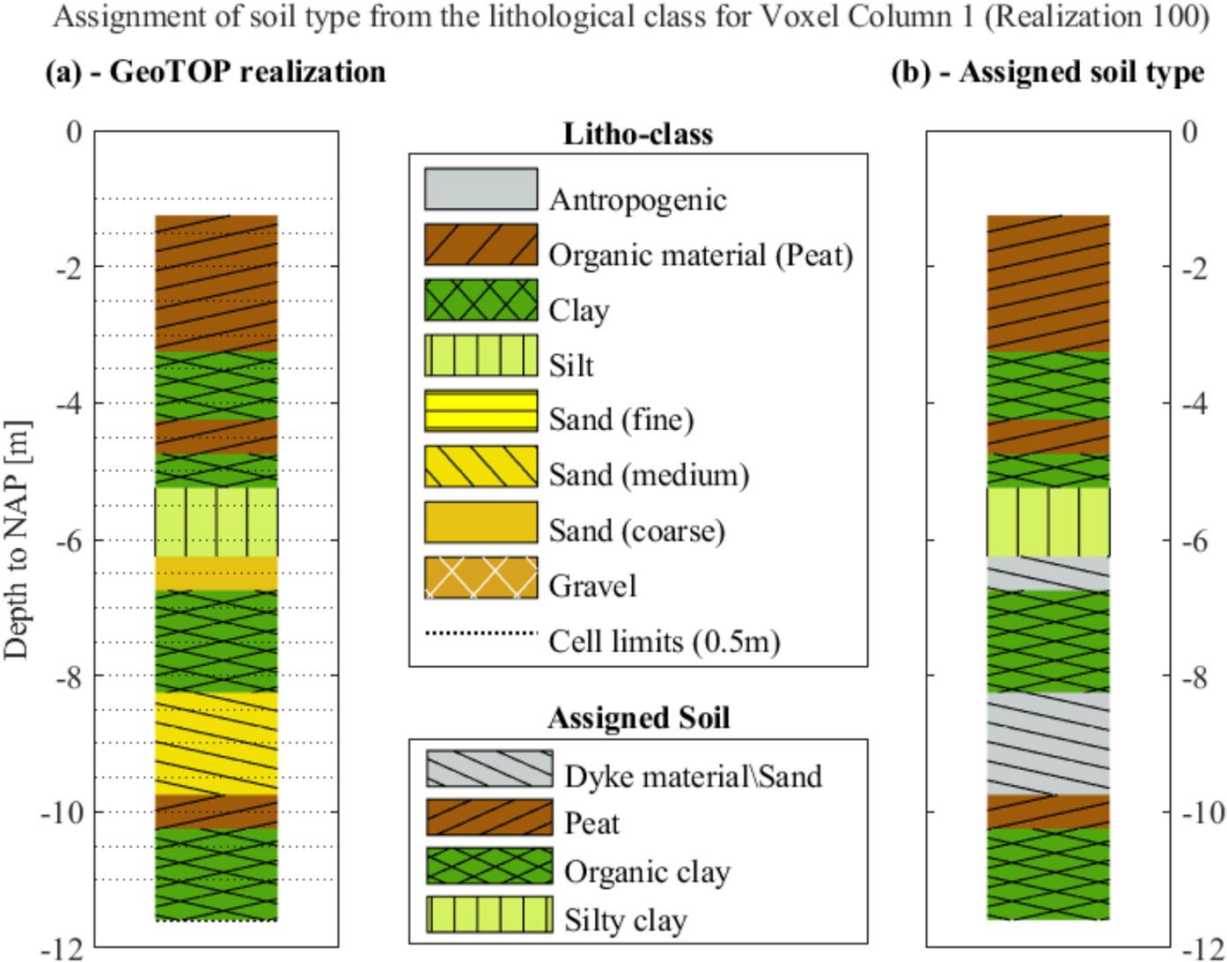
Schematic illustration of the relationship between **a** the computed soil lithoclass from the GeoTOP model and **b** the assigned soil type for each Voxel column realization

The studied area is covered by two Voxel columns (Fig. 3a). 78 CPTs are included in the coverage of Voxel column 1, whereas Voxel column 2 only covers 22 CPTs (Fig. 3a). All the 100 equiprobable realizations for the two available Voxel columns are shown in Fig. 3b and c. Each lithological class was assumed to have the same hydro-geo-mechanical properties as one of the four soil strata in the study area (Table 1), for consistency. This assumption is supported by similar stratigraphic conditions of both the GeoTOP realizations and the CPTs, with superficial strata of peat and clay, and deeper layers of silt and sand. Thus, each litho class is then assumed to have the same characteristics as one soil type for the study area, as shown in Fig. 4.

As briefly mentioned, the “dyke material” consists of sandy and clayey material. Due to that, it was assumed that its behaviour is similar to sand/loam, for the similarity between its material properties reported in Table 1 (de Gast 2020; de Gast et al. 2021a) and those reported in the Dutch norm. Moreover, as briefly mentioned above, the layers of peat and clay, which range between − 2.8 m up to − 10.90 m to NAP, are both characterized by an organic fraction (de Gast 2020; de Gast et al. 2021a). Thus, it is reasonable to assume that soil strata within the same range may be characterized by the presence of an organic fraction. The dyke material properties are thus assigned to the uppermost sand litho-classes of the GeoTOP realizations (Fig. 4), assumed to represent prevalently sandy layers with a small clay/organic fraction that undergoes creep.

In the following numerical simulation, both high-resolution data (from the CPTs) and low-resolution data (from Voxels) are used in separate models, with the results later compared. This approach aims to capture the effects of lithological heterogeneity at different resolutions, facilitating an evaluation of the resulting differences.

### Set-up of the Finite Element Models for Computing Settlements

In this study, 1D, 2D and 3D analyses are carried out with the software PLAXIS 2D and 3D to investigate the effect of in-situ lithological heterogeneity on the settlements occurring at the scale of structures. The two independent datasets available, one high-resolution (from the closely spaced CPTs) and one low-resolution (from the available Voxels), as outlined in Sect. 2.1, are used to create distinct numerical models. The results from these models are then compared. The availability of closely spaced CPTs allows for the use of 1D, 2D, and 3D models to represent the study area, whereas due to the low resolution of the Voxels, which include only two Voxel columns, only 1D analyses could be performed. In particular, the stratigraphic information from the available datasets was used to construct the model geometry, distinguishing the different soil layers.

**Fig. 5:**
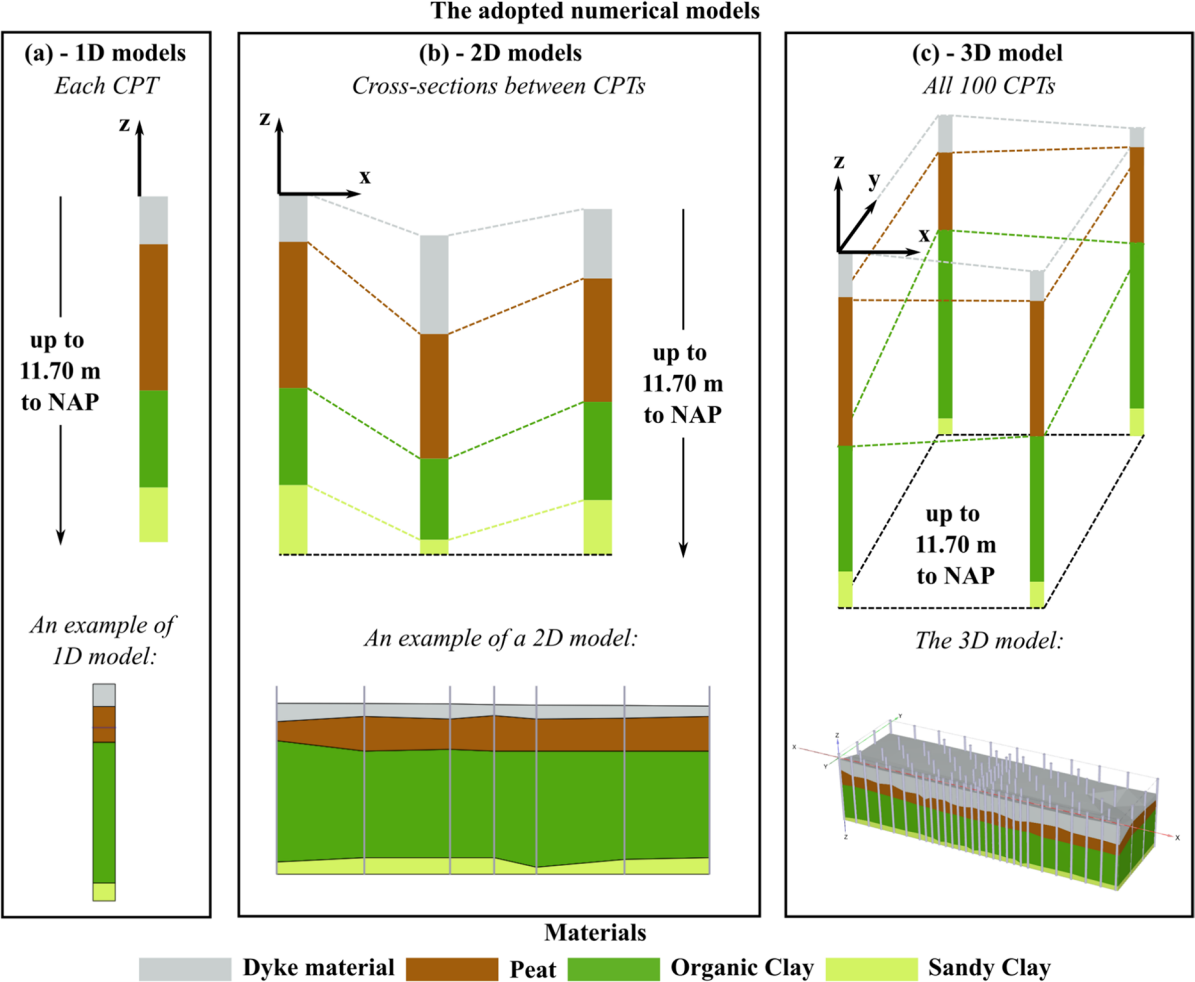
Schematic illustration of the adopted FE models. The directions x and y represent the local axes of the models. z is the vertical axis along the depth direction. The lower boundary at the bottom of the models is schematically illustrated

In the case of the 1D analyses (Fig. 5a), the geometry of the models corresponds to each CPT interpretation (Fig. 2d) and each Voxel column (Fig. 3b and c). It should be noted that all the CPTs were performed from the ground surface, which has a variable elevation in each location as shown in Fig. 2b and c, up to a depth of about 12 m to NAP. Thus, the CPTs are characterized by different lengths along the depth. Therefore, the CPTs were herein truncated at the depth of 11.70 m to NAP (Fig. 5a), which corresponds to the shallowest depth reached among all the CPTs. The mean length of the truncated CPTs, adopted hereafter, corresponds to 10.34 m, while the standard deviation is equal to 0.64 m.

Differently from the CPTs, the elevation of the top level of the Voxel columns does not differ among the 100 realizations. Moreover, as briefly mentioned in Sect. 2.1, the Voxel columns provide the stratigraphy up to a depth of 50 m to NAP. In this study, however, only the portion of the Voxel columns that covers the depth of the study area is considered. Therefore, each Voxel column is truncated in a way that ensures a length of 10.34 m (Fig. 3b and c), equal to the average length among all the CPTs. The 2D models represent the cross-sections obtained by interpolating the stratigraphic information of the available CPTs (Fig. 5b) in the directions that correspond to the short and long sides of the CPTs’ field (directions 1 and 2 in Fig. 2c respectively). The same procedure is applied to obtain the 3D model (Fig. 5c).

The practical advantage of truncating the CPTs at the depth of 11.70 m consists of having a horizontal boundary at the bottom of the 2D and 3D models (Fig. 5b and c). Using a horizontal edge in the 1D and 2D models, and a horizontal surface in the 3D models, facilitates the creation of a mesh with more regularly shaped elements. This approach helps prevent issues related to irregular elements, which could potentially cause the numerical analyses to diverge.

In all the models, a constant groundwater table of − 3.94 m to NAP is used, idealizing the in-situ conditions during the time the CPTs were made. The imposed groundwater level is different from the current in-situ conditions, as part of the study area is now submerged, as shown in Fig. 3a.

**Fig. 6:**
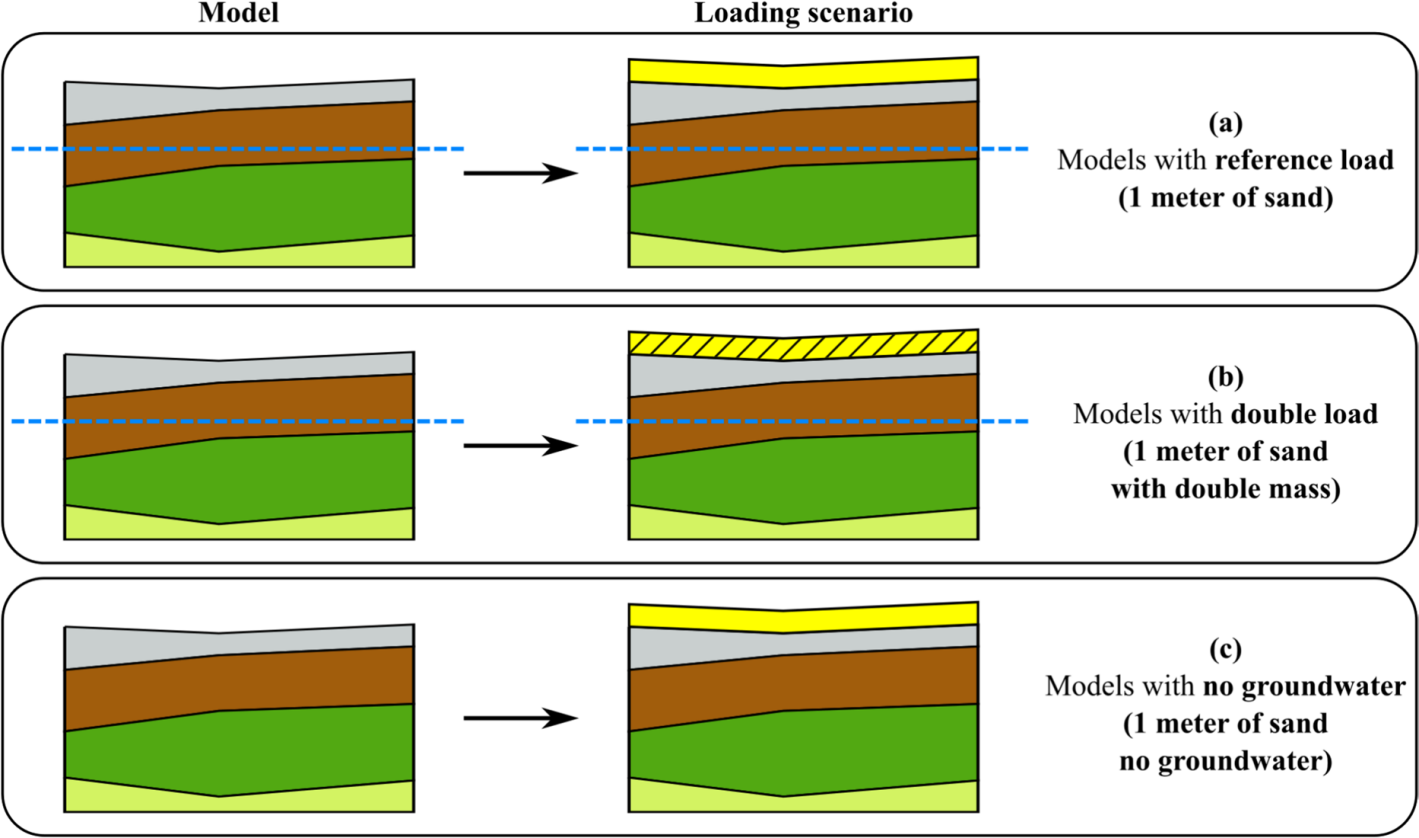
Schematic illustration of the three load scenarios for the 1D, 2D and 3D models

For both 1D and 2D analyses, 15-node triangle plane strain elements are used to discretize the geometry, whereas the 3D models make use of 10-node tetrahedral elements. Meshing is performed by setting the global coarseness factor equal to 0.07 in all the models. For additional information on the coarseness factor and its influence on the element size, the reader is referred to (Bentley 2023). The soil layers are modelled with the “Soft Soil Creep” model (SSC), using material properties reported in Table 1. The SSC model is able to account for the compressive behaviour and viscous effects of very soft soils and requires three stiffness parameters: the modified swelling index κ*, the modified compression index λ* and the modified creep index µ*. SSC is also assigned to the “dyke material” for its clay fraction. In all the models, a constant load that simulates the deposition of 1 m of sand with a volumetric weight of 17 kN/m^3^ is applied on the surface to trigger the consolidation process (Fig. 6a). The additional load of 1 m of sand represents a hypothetical scenario that helps compare the sensitivity of different sections of the case study to settlement. This load allows for an evaluation of how various locations within the study area react to settlement, highlighting the differences in their responsiveness to the applied weight, following an approach similar to (Erkens et al. 2021). In the Netherlands, urban development on soft soils often involves adding sand to the surface layers to compress the underlying soft soil before constructing new buildings. This sand addition simulates this process, although, in practice, the actual sand thickness may be different. This loading condition is herein labelled as “reference load” (Fig. 6a). For the sensitivity study, additional analyses are carried out by doubling the reference load (“double load” in Fig. 6b) to amplify the plastic deformation in the model. Another set of analyses uses the reference load but the groundwater table was completely removed (“no groundwater” in Fig. 6c) by fictitiously setting the water head to − 15 m to NAP in the models. This adjustment eliminated consolidation effects, allowing for further evaluation of the groundwater table’s influence on settlement behaviour.

The adopted finite element models represent hydro-mechanical models, which account for the interaction between the hydraulic and mechanical behaviour. A phased analysis approach is employed in all the models, simulating sequential loading stages to study the progressive behaviour of the soil as it evolves over time. Therefore, the generation of the initial stresses of the soil, the application of the load on the ground surface resulting in the generation of excess pore water pressure and the consequential consolidation process are distinguished in the analysis as follows:

1. First, the pore pressure and the stresses are initialized using the K0 procedure. This procedure defines the initial stresses taking into account the loading history of the soil (Bentley 2023). The relationship between the vertical and horizontal stresses is determined by the coefficient K0, as a function of the pre-overburden pressure (POP in Table 2).
2. Then, the load is applied in the Plastic phase, and the excess pore water pressure is generated and has no time to dissipate.
3. Finally, consolidation phases are used to simulate the consolidation process at different time intervals. A consolidation calculation is typically performed to evaluate the development and dissipation of excess pore pressure over time (Bentley 2023). To avoid potential iteration convergence issues, load application and consolidation are addressed separately instead of applying the load directly via a consolidation analysis (Bentley 2023). This phase is a time-dependent phase and simulates the consolidation process and creep.

**Table 2:** The adopted phased analyses and numerical settings

Phase	Calculation type	Pore pressure calculation time	Time [days]	Max steps	Tolerated error	Max number of iterations	Max load fraction per step
Initial phase	K0 procedure	–	–	–	–	–	–
Load application	Plastic	Use pressures from the previous phase	0	1000	0.01	60	0.5
From 1 up to 5000 years of consolidation (1, 10, 20, 30, 40, 50, 100, 500, 5000 years)	Consolidation		From 365 up to 1.825 × 10^6^				

Additional information regarding the different settings of phased analyses used for the numerical analyses is summarized in Table 2. Accordingly, the settings of the iteration procedure are reported.

Regarding the boundary conditions of the models, During the K0 procedure, the initial water pressure is hydrostatic and based on the groundwater level. In this phase, all boundaries except for the bottom boundary are draining. During the load application and consolidation phases, the drainage boundaries are assumed to be at the ground surface and the bottom of the soil, while the lateral boundaries are considered closed. The horizontal displacements at the lateral boundaries are fixed while the vertical displacements are free (de Gast 2020).

Settlements, defined as the time-dependent reduction in height of each soil layer (or the cumulative sum of their contributions), are computed using finite element analyses.

### Correlation Length for Spatial Variability of Settlement and Soil Lithological Heterogeneity

The correlation length or scale of fluctuation, herein labelled as “$\theta$”, represents a convenient metric to describe the distance within which observations are significantly correlated (Vanmarcke 1984). The correlation length $\theta$ is determined by fitting the empirical determined auto-correlation function $\widehat{\rho }$ described in Eq. (1):

1 $$\widehat{\rho }=\frac{\widehat{\gamma }(\tau )}{\widehat{\gamma }(0)}$$

where $\widehat{\gamma }(\tau )$ represents the empirical covariance function for the lag distance $\tau$. For observations unevenly spaced on a grid, such as the grid represented by CPTs’ locations of the study area herein presented, $\widehat{\gamma }(\tau )$ can be computed as (2) (de Gast 2020):

2 $$\widehat{\gamma }\left(\tau \right)=\frac{1}{t-1}\sum_{j=1}^{t}({y}_{j}-\widehat{\mu })({y}_{j+\Delta j}-\widehat{\mu })$$

where $\widehat{\mu }$ is the mean of the dataset, $j$ is a counter representing the index of the first of a pair at lag distance $\tau$, $\Delta j$ represents the index spacing of a specific pair of observations for a non-uniformly distributed dataset and $t$ is the number of pairs at lag distance $\tau$. The lag distance represents the physical distance between the observations in the dataset.

In other words, for each lag distance $\tau$, a sub-sample is defined as the number of pairs of observations $t$ at that distance $\tau$. The covariance is $\widehat{\gamma }\left(\tau \right)$ is then computed for the sub-sample, using the mean of the entire dataset $\widehat{\mu }$.

The empirically determined auto-correlation function $\widehat{\rho }$ is fitted by the squared-exponential (Gaussian) function in Eq. (3):

3 $$\rho (\tau )=exp\left[-\pi {\left(\frac{|\tau |}{\theta }\right)}^{2}\right]$$

A MATLAB algorithm is used to perform the fitting procedure to obtain the correlation length $\theta$. In particular, data are grouped for intervals of lag distances $\tau$ of 1 m. A lower bound of $\theta$ is imposed equal to the minimum distance between two CPTs of the study area (about 1 m in this study). The lower bound is imposed to avoid values of $\theta$ smaller than the minimum distance between CPTs, which would have no physical meaning.

## Results

### Quantification of Local Lithological Heterogeneity Using the Correlation Length for Variable Soil Thicknesses

**Fig. 7:**
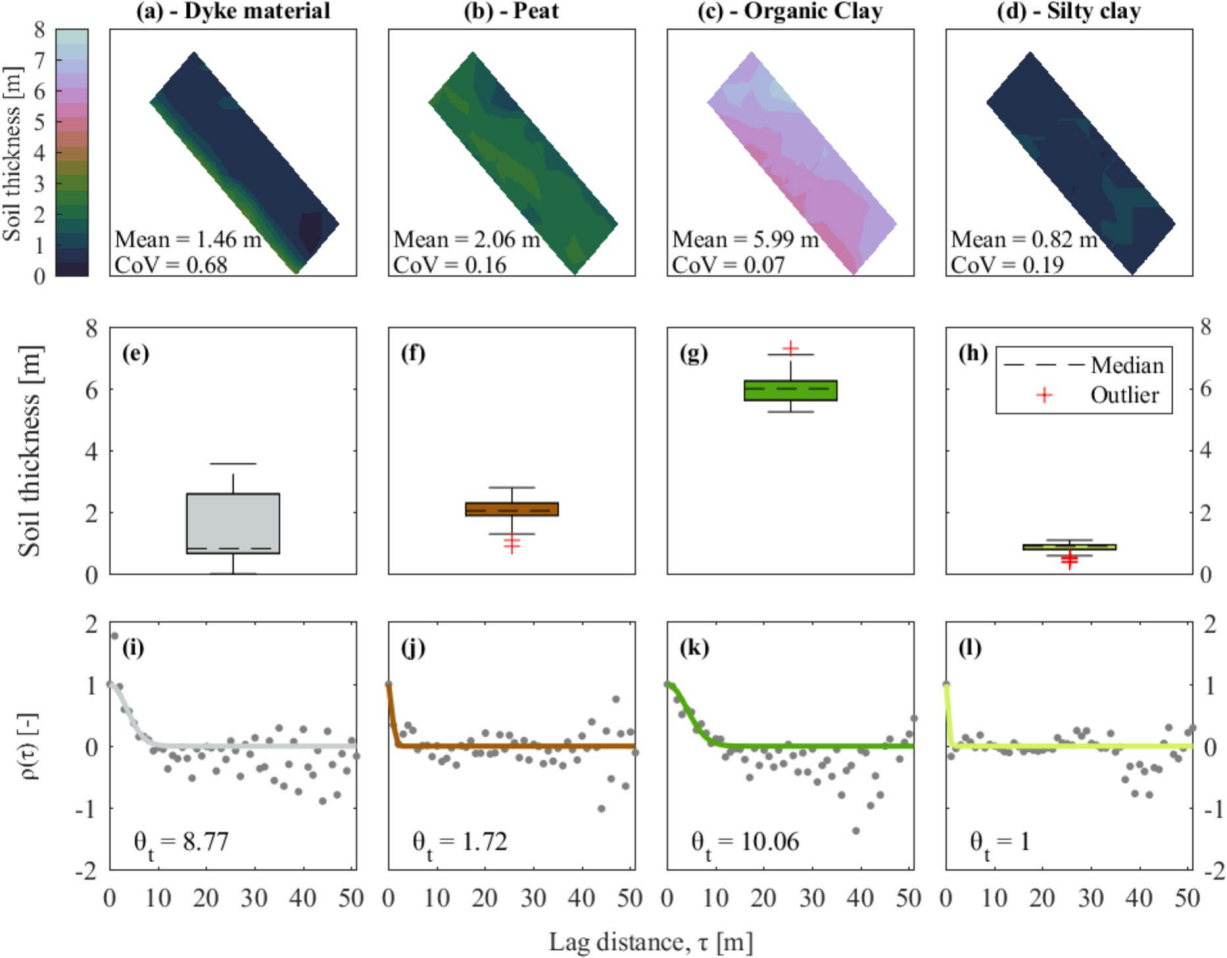
Soil layer thickness maps (top row), box plots (middle row), and autocorrelation analyses (bottom row) for different soil materials: **a**, **e**, **i** Dyke material, **b**, **f**, **j** Peat, **c**, **g**, **k** Organic clay, and **d**, **h**, **l** Silty clay. The maps in (**a**–**d**) are obtained via linear interpolation and display the spatial variation in soil thickness. The box plots in (**e**–**h**) show the distribution of soil thickness values, highlighting medians and outliers. The autocorrelation plots (**i**–**l**) present the empirical autocorrelation function ρ(τ) (grey dots) and the fitted function (solid line), with the corresponding correlation length θ_t_ ​ provided for each soil material

The maps in Fig. 7a–d display the spatial distribution of the soil thickness for each soil material. The thickness of the dyke material is higher, as expected, in proximity to the dyke crest, whereas the thickness of the organic clay progressively increases toward the polder. The organic clay material exhibits, on average, the highest thickness (5.99 m), see Fig. 2, followed by the peat (2.06 m) the dyke material (1.46 m) and the silty clay (0.82 m). The dispersion of the values of soil thickness is reported using the Coefficient of Variation (CoV) in Fig. 7a–d. The organic clay layer is associated with the smallest dispersion (0.07) followed by the peat (0.16), the silty clay (0.19) and the dyke material (0.68).

The variation of the soil thickness for each layer is visualized using box plots in Figs. 7e to h, which display the median (dashed lines), the interquartile range (the height of each rectangle) and the upper and lower bounds of the thickness of the soil strata of the available CPTs logs.

The soil thickness at each CPT location is used to determine the correlation length of the material thickness, labelled as θ_t_, for each material (Fig. 7i–l), according to the procedure detailed in Sect. 2. The dots in the plots represent the values of $\widehat{\rho }$ obtained with Eq. (1), while the lines result from the fitting procedure that uses Eq. (3).

The organic clay is the soil strata associated with the highest value of the θ_t_, followed by the dyke material, the peat and silty clay layer. However, the silty clay presents a θ_t_ equal to the lower boundary imposed in the fitting procedure, i.e., 1 m. This can be related to the fact that the silty clay has a limited (and truncated, as briefly described in Sect. 2.2) thickness. For this reason, the computed values of the correlation length for the silty clay may be less reliable compared to those for the other soil layers.

### Correlation Length of the Computed Settlements at In-Situ and National-Level Resolutions

**Fig. 8:**
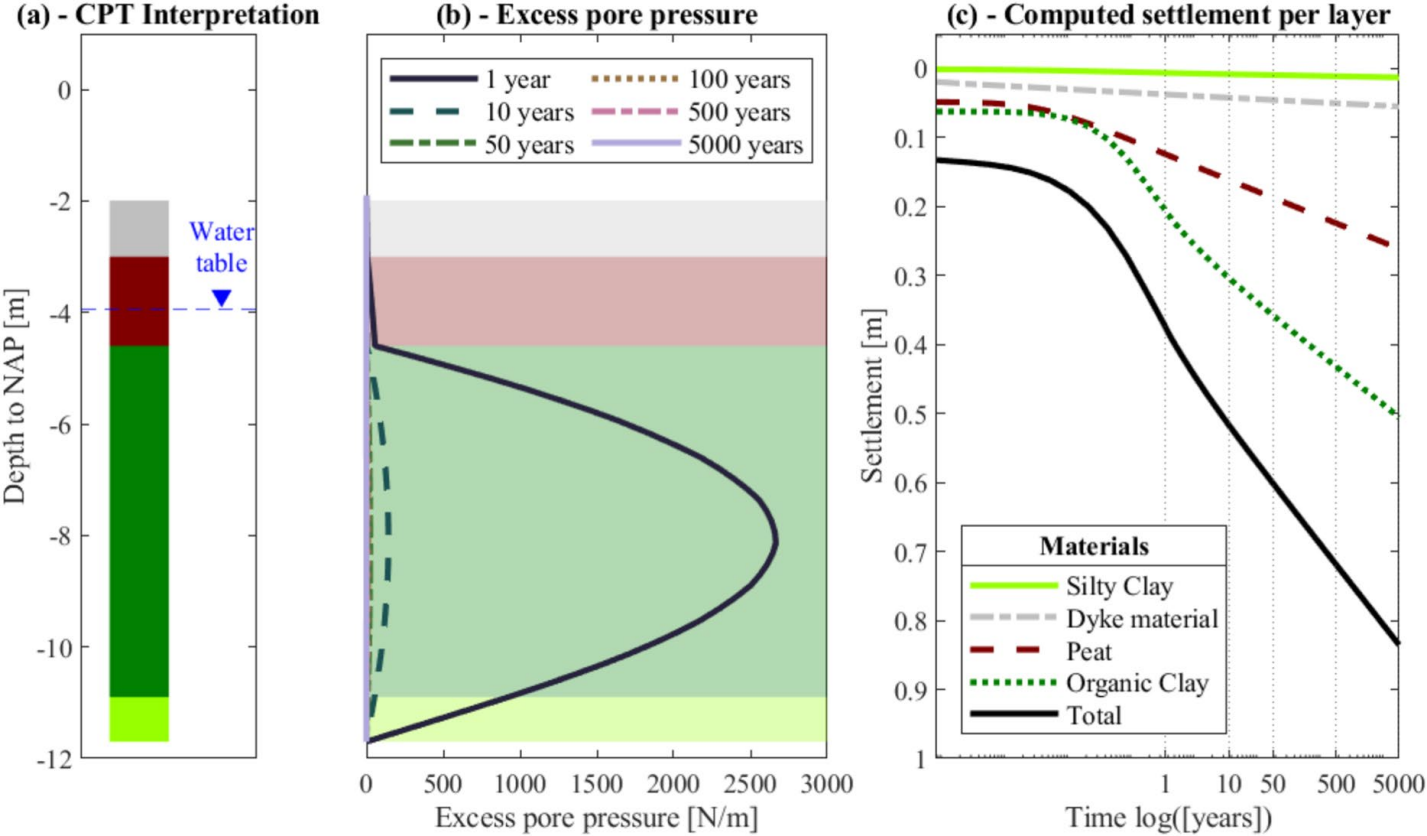
An example of the results of one of the 1D models subjected to the reference load: **a** the CPT interpretation and the groundwater level, **b** the development of the excess pore water pressure and **c** the computed settlements against time

The numerical models give the vertical displacements of each soil stratum (i.e., dyke material, peat, organic clay, silty clay), and the cumulative vertical displacement, i.e., the sum of all the contributions corresponding to the settlement of the ground surface, at the location of each CPT, for both the 1D, 2D and 3D analyses. In the case of the Voxel columns, the material strata at a certain depth vary among the different realizations, thus it is not possible to retrieve the displacement of each material and only the cumulative vertical displacement is considered. An example of the results of the 1D numerical analyses is shown in Fig. 8 for the reference load. The organic clay material is the layer where the excess pore pressures develop the most (Fig. 8b) and, in turn, it is the layer that contributes the most to the total settlement (Fig. 8c). On the contrary, as expected, the silty clay has the smallest contribution to the total settlement (Fig. 8c), which is also the result of its small thickness (Fig. 7d and h) and the low compressibility assigned (Table 1). For this specific model, the excess pore pressure (Fig. 8b) is observed to dissipate rapidly, within less than 10 years. The settlement observed thereafter is primarily the result of secondary consolidation of the peat and organic clay layers (Fig. 8c). This aspect is evident as the settlement versus the logarithm of time curves change the slope and become linear when the creep effect becomes predominant (Fig. 8c). As a consequence, the final settlement, after 5000 years, will be primarily influenced by creep, once the excess pore water pressure has been fully dissipated at all CPT locations.

**Fig. 9:**
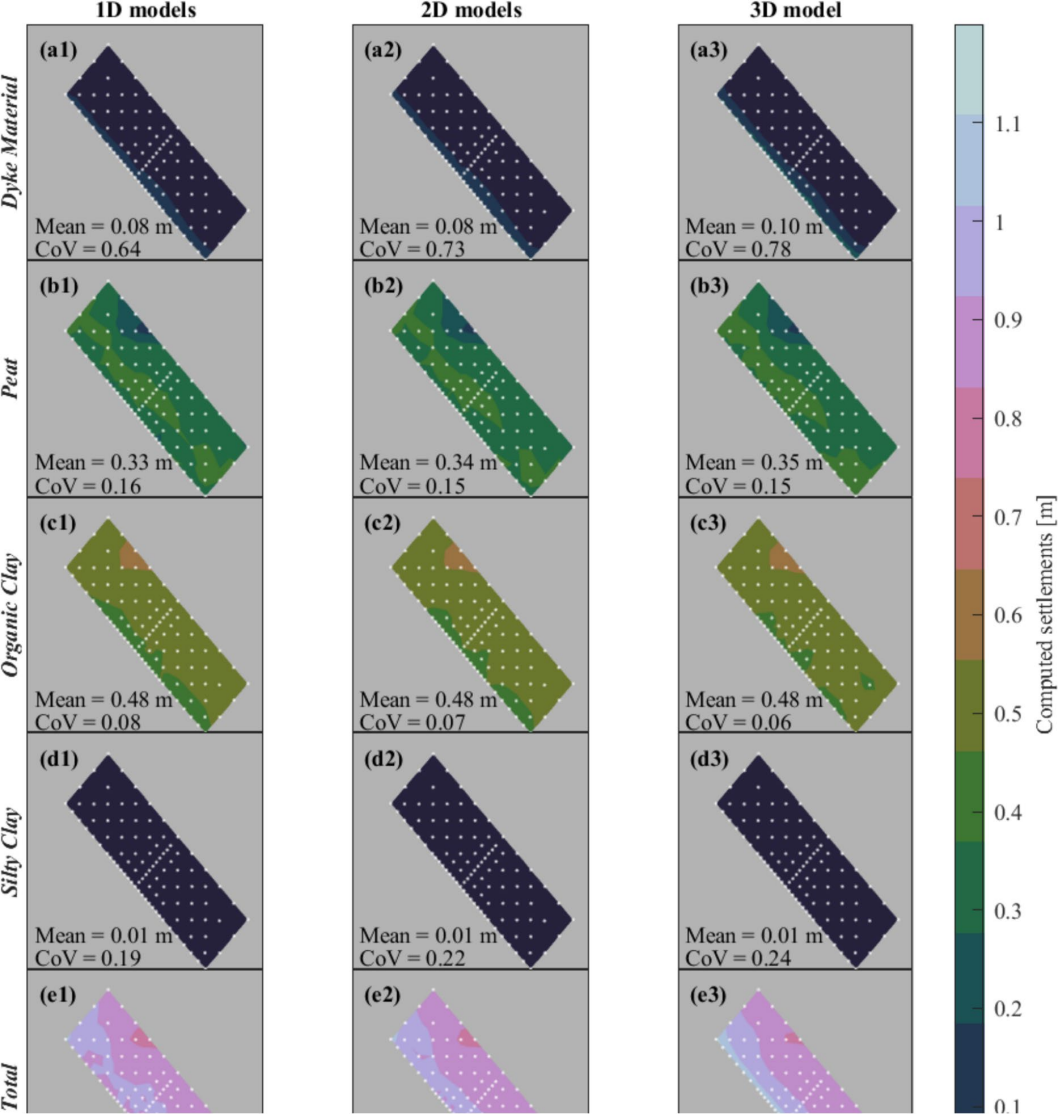
The settlement maps are derived from the linear interpolation of the final (i.e., 5000 years) settlement computed at each CPT location via the numerical models of the reference load (Fig. 6)

A comprehensive picture of the effect of the different soil layers is shown in Fig. 9. Accordingly, the final (i.e., 5000 years) settlement of each layer is shown with maps obtained by linearly interpolating the results of the 1D, 2D and 3D models subjected to the reference load at all the CPTs’ locations. The maps provide a picture of the spatial variability of the computed settlement for each soil layer and the sum of their contributions. The results confirm how the layers “dyke material” (Fig. 9a1–a3) and “silty clay” (Fig. 9d1–d3) contribute the least to the overall settlements at all the CPTs’ locations, as observed in Fig. 8. As expected, for each soil stratum, the areas with the highest and lowest settlements correspond to those of the soil thickness specific to that material (shown in Fig. 7). In other words, the spatial variability of the soil thickness matches that of the computed settlements for each material. Moreover, the values of the dispersion, i.e. CoV, of the computed settlements for each material (Fig. 9) match those of the material thickness for each soil (Fig. 7). In particular, the organic clay layer is associated with the smallest dispersion (from 0.06 to 0.08) followed by the peat (0.15 and 0.16), the silty clay (from 0.19 to 0.24) and the dyke material (from 0.64 to 0.78).

The similarity in the CoV of the computed settlements for each soil layer and the corresponding CoV of the material thickness suggests that their variability is comparable, despite the CoVs referring to different metrics.

Each layer is observed to have settlements that only slightly change in terms of magnitude and spatial distribution among the 1D, 2D and 3D analyses. Nevertheless, the differences among the layers accumulate, and the total settlement varies among the analyses considered (Fig. 9e1–e3). While it appears that the spatial variability of settlement across the soil layers is not influenced by the 1D, 2D, or 3D numerical models, the sum of their contributions suggests otherwise.

In Fig. 9e3, it is observable how the 3D analyses better distinguish the behaviour of the three portions of the dyke, i.e., the crest, slope and polder (Fig. 2b), whereas the settlement is more uniform in the case of 1D (Fig. 9e1) and 2D models (Fig. 9e2).

The computed vertical displacements of each soil stratum (shown for instance in Fig. 8) are used to obtain the correlation length of the settlement θ_s_ for five time steps, 1, 10, 50, 500 and 5000 years. Hence, the use of five time steps allows investigation of the time dependency of θ_s_.

**Fig. 10:**
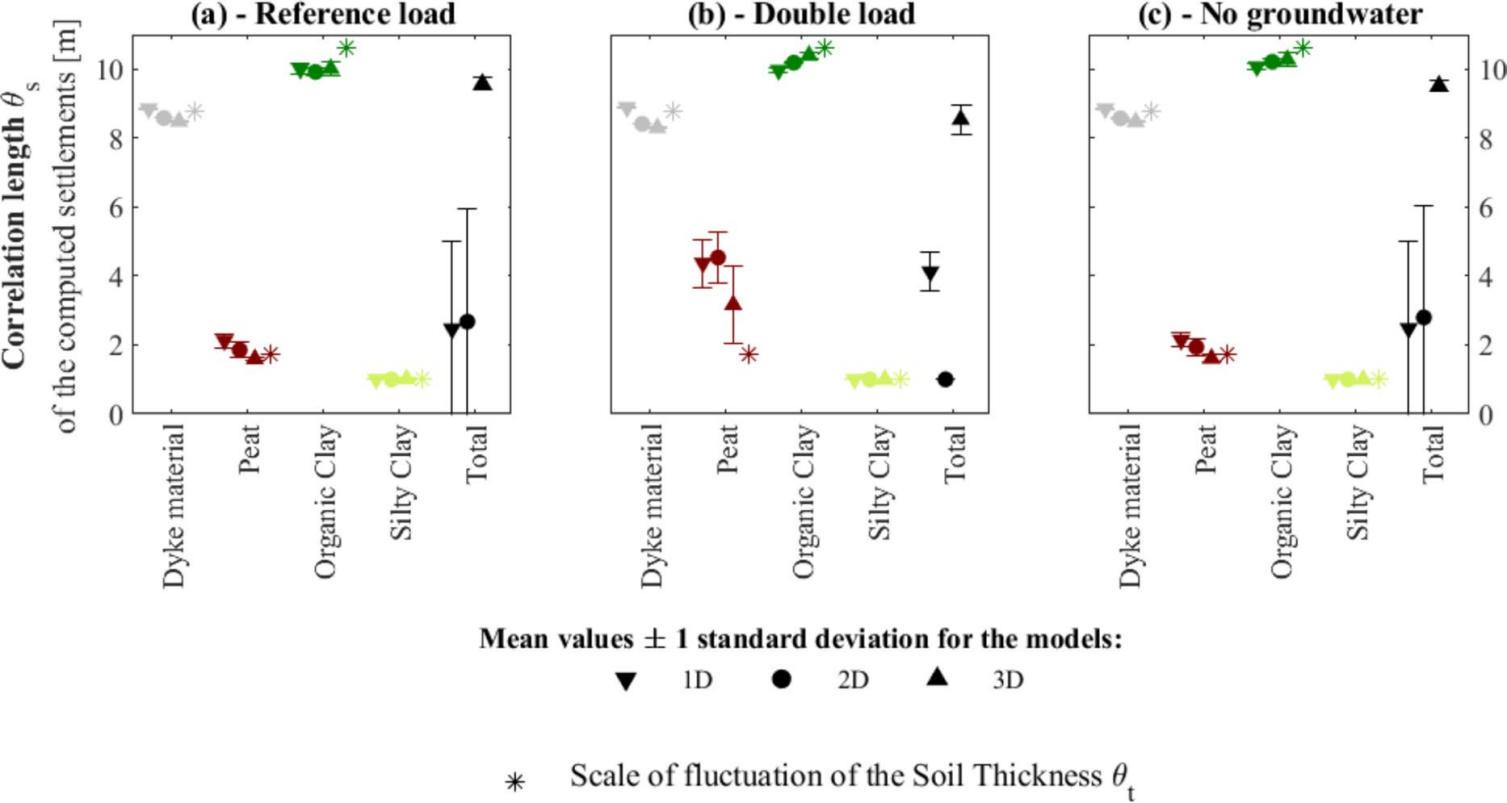
Values of the correlation length of the computed settlement of each soil statum for each loading condition. The values correspond to the mean and the standard deviation of θ_s_ considering the five time steps, i.e., 1, 10, 50, 500 and 5000 years for: **a** reference load, **b** double load and **c** no groundwater (see Fig. 6)

Figure 10 shows the mean (markers) and the standard deviation interval (error bars) of the values of θ_s_ for each soil layer and of the total settlement, computed considering the selected time steps. The plots are distinguished by the type of loading scenario (Fig. 6). Additionally, the values of the correlation length of the soil thickness θ_t_ for each material, already reported in Fig. 7, are also plotted to allow a better visual comparison between the results.

The type of analyses (i.e., 1D, 2D and 3D models) is not observed to significantly influence the values of θ_s_. This can be attributed to the above-mentioned similarity between the spatial distribution of the computed settlements for each layer, shown in Fig. 9. However, the small localized differences in the computed settlement of each layer influence the spatial distribution of the total settlement, hence influencing its θ_s_ values.

In the case of the peat layer, the scale of fluctuation θ_s_ varies depending on the considered loading scenario: in the case of the double load, the peat layer exhibits higher mean and standard deviation values of θ_s_ for all the types of models. This is explained by the fact that in the case of the double load, the peat layer has a higher contribution to the overall settlements at some specific CPT locations compared to the case of the reference load and the models with no groundwater. This can be a consequence of the low compressibility assigned to the peat layer (Table 1), which makes it the stratum that is influenced the most by a change in the applied superficial load. Additionally, the observed time dependency may stem from the fact that some locations experience settlement more rapidly than others, thereby amplifying the scale of fluctuation in the peat layer.

The values of θ_t_ and θ_s_ are observed to be in good agreement for all the soil strata.

### Comparison Between In-Situ (CPTs) and National-Level (Voxel Columns) Models

**Fig. 11:**
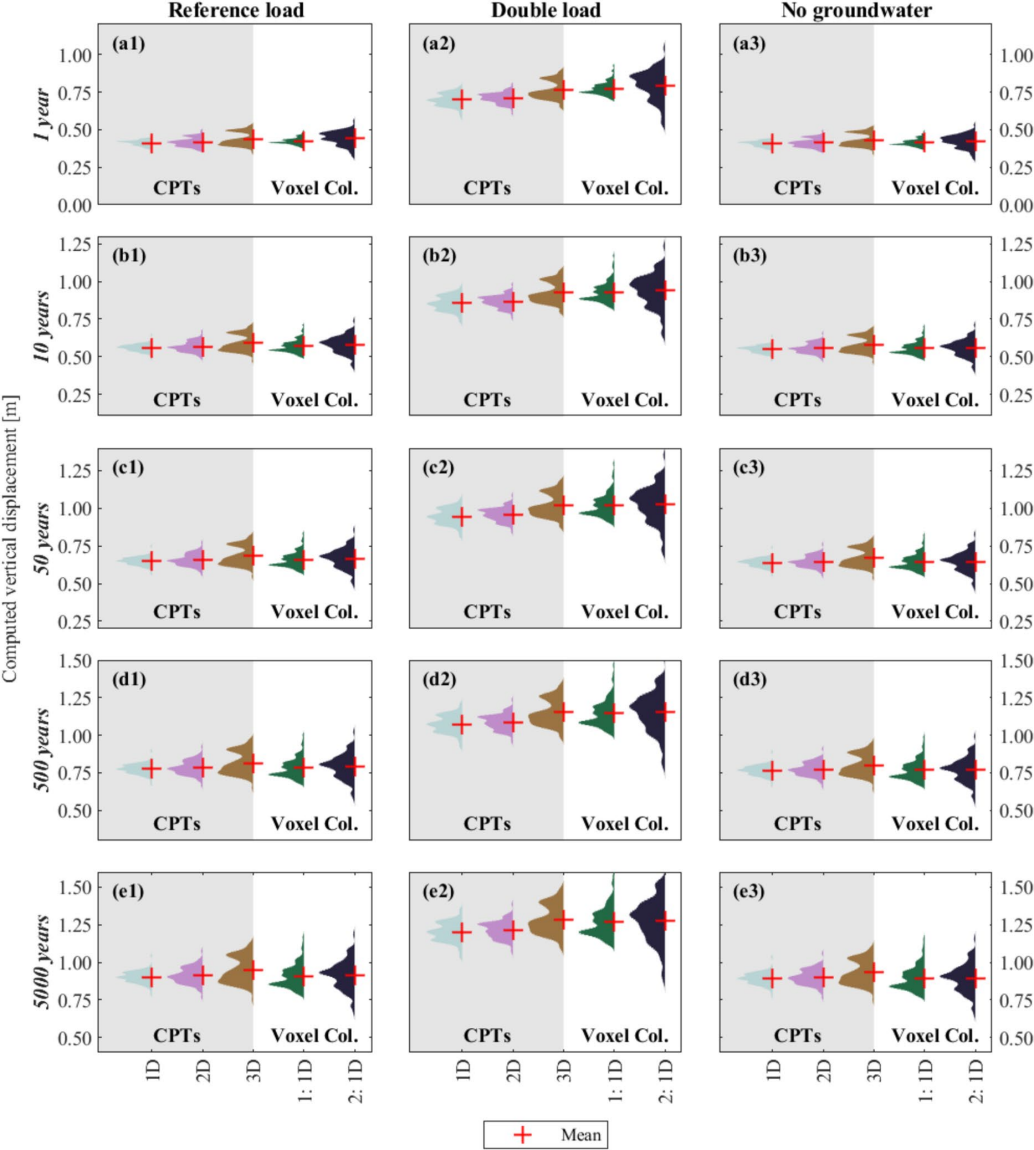
Empirical distribution functions of the total settlement computed for all the loading conditions and the numerical models, described in Sect. 2.2, for the selected time steps. The distributions show the results at the 100 CPTs’ locations (Fig. 2) and the 100 realizations for two Voxel columns (Fig. 3). The darker and lighter areas in the plots separate the results for the CPTs and the Voxel columns, respectively. The red cross (+) markers show the mean of each distribution

The settlement of the in-situ specific 1D, 2D and 3D models and the 1D Voxel realizations are plotted as distributions to investigate their differences (Fig. 11). The distributions are shown for each analysis, loading condition and selected time step. Additionally, the values of the mean and coefficient of variation of the distributions are plotted and they are reported in Table 3.

**Table 3:**
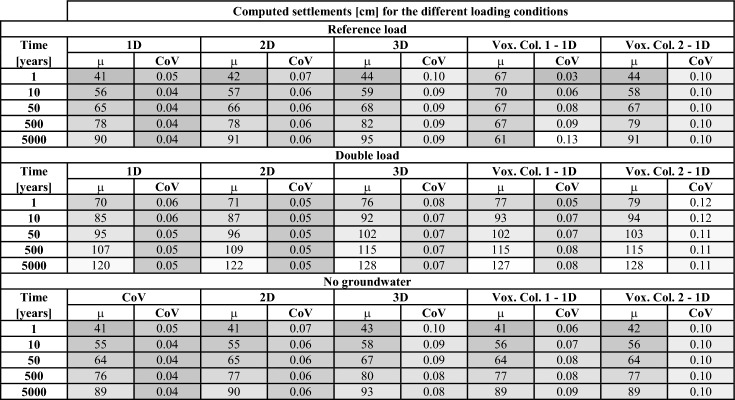
Mean (μ) and coefficient of variation (CoV) values of the computed settlement at the surface level for all the numerical analyses and all the loading conditions. A colour scheme is applied to each parameter, i.e. μ and CoV, to distinguish the lowest (dark shades) and the highest (light shades) values

Although for each loading scenario, the results of all the models have similar values of mean and dispersion (Table 3), their shapes slightly vary: the distribution of the computed settlement of the 3D models resembles a non-symmetric bi-modal distribution, due to the difference between the results of the dyke crest and slope, and the polder area. Conversely, the 1D and 2D models are more akin to unimodal distributions.

The results reported in Table 3 confirm that overall, 1D, 2D, and 3D analyses based on the high-resolution dataset do not show major differences in terms of mean or dispersion. However, the CoV consistently increases from the 1D analyses to the 2D, and then further to the 3D models, while the changes over time are negligible. In particular, the 3D models have twice the relative variability of the 1D analyses, and this aspect indicates greater heterogeneity in the computed settlements. As discussed in Sect. 3.2, 3D analyses may more accurately capture the variable response across different locations of the study area compared to 1D and 2D analyses.

The 1D models of the Voxel columns present values of the mean and dispersion similar to those of the 1D, 2D, and 3D in-situ specific analyses (Table 3). In particular, the computed settlement of Voxel Column 1, which covers most of the study area (Fig. 3a), presents a bi-modal shape similar to the 3D in-situ specific models. However, the variability in the results for Voxel Column 1 can primarily be attributed to the variability in the stratigraphy, which is inherent to the low-resolution dataset (see Sect. 2.1) and does not necessarily reflect the realistic variability of the study area’s features. Nevertheless, the similarity in terms of mean and dispersion of the computed settlement between the Voxel columns (low-resolution dataset) and the CPT-based models (high-resolution dataset) suggests a good agreement in the representation of the overall response of the study area between the two datasets.

The agreement between the CPT-based and Voxels models is not significantly influenced by the different load scenarios. Depending on the loading scenarios, the magnitude of the mean and dispersion of the computed settlement may vary, however, low-resolution models present good agreement with the high-resolution models.

## Discussion

In this study, an area is selected to investigate lithological heterogeneity and its effects on settlement occurrence. Toward this aim, exploratory analyses are carried out by means of finite element models, including 1D, 2D and 3D numerical analyses and different loading scenarios. The loading conditions represent idealizations, they are not intended to reproduce realistic consolidation scenarios for the study area. Conversely, they enable investigation of how lithological heterogeneity influences the spatial variation of the computed settlement due to different imposed loads. It is important to note that the findings of this study could be significantly enhanced through further research, particularly validation against experimental or field data. However, such data are not currently available.

It should be noted that this study mainly focuses on the effects of soil stratification, while more complex loading conditions may further enhance the spatial variation of the computed ground settlements, as reported in (Peduto et al. 2022).

In the selected study area, no structures exist on the ground surface. The presence of subsurface structures, such as residential buildings or embankments, could also add complexity to the problem and has been intentionally disregarded in this study. In other words, this research excludes the impact of structures and instead focuses on the variability in soil strata thickness. Nevertheless, such effects should be considered in future research.

Moreover, the effects of the variability of the hydro-geo-mechanical properties, i.e., inherent spatial soil variability, are purposively neglected to focus on the sole impact of lithological heterogeneity.

### Quantification of Soil Thickness Variability and Comparison with Prior Research

Regarding the scale of fluctuation of the material properties, (Breysse et al. 2005) reported there is always a critical value that can augment differential settlements, which depends on the governing geometrical parameters and conditions of the considered problem. Furthermore, prior research has demonstrated how variations in soil properties beneath foundations can exacerbate distortions and lead to differential settlements that affect structures (Houy et al. 2005; Denis et al. 2011; Jamshidi Chenari et al. 2019; Bezih et al. 2025). However, previous studies often focus on the impact on settlement without distinguishing whether the variations in soil properties beneath structures are primarily due to changes in mechanical and hydrological properties or variations in stratigraphy. The analyses reported in (Jamshidi Chenari et al. 2019) show that the differential settlement of the adjacent footings bears greater values for the scales of fluctuation ranging from 2 to 6 m. Similarly, a critical correlation distance ranging from 4 to 12 m has been identified in (Bezih et al. 2025) for reinforced concrete structures.

The results of this study suggest that the combination of both the spatial variability of lithological conditions and material properties could potentially enhance differential settlements more than either factor alone.

This study focused on the spatial variability of the lithological conditions, including the soil types and their thickness, rather than the material properties. However, the computed values of the correlation length θ_t_ (Fig. 7) range between about 2 and 10 m (Fig. 7). Therefore, even though the correlation length addressed herein refers to lithological heterogeneity rather than the variability of the material properties, the values align with those reported in (Jamshidi Chenari et al. 2019; Bezih et al. 2025).

These findings have implications for the design of foundations and structures: for example, the results emphasize the potential risks of assuming that soil strata are uniformly horizontal, with no variation in thickness or inclination. While such an assumption may serve as a useful simplification for many engineering problems, it should be applied with caution, as it may not accurately capture the complexities of the actual subsurface conditions.

### The Variability of the Computed Settlement in High-Resolution Models

The in-situ specific 1D, 2D and 3D numerical simulations enable computing the contribution of each soil layer to the total settlement, and their spatial variability θ_s_, as shown in Fig. 10. The computed values of the correlation length θ_s_ of all the soil layers do not depend on the groundwater conditions. In the case of the peat layer, the values of θ_s_ are observed to vary at different time steps of the model subjected to the doubled load.

For all the soil layers, a good match is observed between the values of θ_t_ and θ_s_ (Fig. 10), pointing toward a relationship between the two parameters. In other words, the analyses reveal that spatial variability of the material thickness of each soil layer matches the one of their contribution to settlements.

In particular, the dispersion (CoV) and spatial variability (θ) of the material thickness are observed to mirror those of the computed settlements, as observed comparing Figs. 7 and 9. This supports the idea that lithological heterogeneity may directly influence the occurrence of differential settlements at the scale of structures, as often seen in practical scenarios, and, in turn, lead to damage. Further studies that include the presence of the building and its response are recommended to test this conclusion.

The correlation length θ_s_ of the total settlement changes depending on the type of model, i.e., 1D, 2D or 3D (Fig. 10). In particular, the 3D subsurface models present the highest values with the smallest dispersion of θ_s_. Moreover, 1D models in terms of total settlement (Fig. 9a3) are visually more uniform and present less variation compared to the results of the 2D and 3D models (Fig. 9a3–e3 respectively). This trend is confirmed by the distributions of the computed settlement, plotted in Fig. 11. In general, 3D models better represent the interaction between the different CPTs, compared to 2D in which the interaction is limited to one of the directions (i.e., x-axis in Fig. 5) and 1D models, in which it is excluded. Although in all the analyses the drainage of the excess pore water pressure is allowed only on the top and bottom of the numerical models, as briefly described in Sect. 2.2, the excess pore water varies in the x-direction (Fig. 5b) in the 2D models, and in the x- and y- direction in the 3D models (Fig. 5c). Thus, this leads to the development of different stresses and, in turn, to the difference in the computed settlements. The 3D models better distinguish the behaviour expected from the different areas of the dyke, as described in Sect. 3, and are thus associated with the greatest heterogeneity in the computed settlements. This observation supports the use of 3D analyses to accurately depict the influence of lithological heterogeneity on similar problems. However, it is important to note that 3D analyses generally involve greater model complexity and computational demands compared to 1D and 2D models, which may limit their broader application. 3D models often necessitate detailed stratigraphic information, which is readily available in this study. However, in other applications where such detailed data is limited or unavailable, the use of 1D or 2D analyses may become a practical necessity.

### Comparison Between the In-Situ and National-Level Models: The Effect of Different Resolutions

The distributions of the computed settlements of the 3D models resemble asymmetric bi-modal distributions for all the loading conditions and all the selected time steps (Fig. 11). This effect, as mentioned above, is related to the different responses of the dyke crest, slope and polder. A similar shape is observed in the case of Voxel column 1. Voxel column 1 may better idealize the in-situ subsoil conditions compared to Voxel column 2, as it covers the same area covered by the majority of the CPTs (Sect. 2.1). Therefore, the similarities in terms of the shape of the distribution, mean values and standard deviation between the results of the CPTs’ models and the Voxel models (Table 3) may indicate that both resolutions can depict the variability of the settlement in the study area. In other words, the 100 equi-probable realizations of the GeoTOP model at a specific location may provide a picture of the variability of the lithological conditions and, in turn, the variability of the settlement. However, it is important to mention that the 100 realizations differ not only as a result of the soil heterogeneity at a specific area, but also due to the modelling assumptions used to derive them, as well as the adopted borehole logs, their number and quality (Stafleu et al. 2011, 2021). Thus, although in this study the variability of the settlements computed at the scale of single structures is reflected by the variability of the settlements of the Voxel realizations for the study area (see Fig. 11), additional analyses are required to further validate this conclusion in other areas.

The differences between low-resolution (Voxels) and high-resolution (CPTs) models are not observed to depend on the loading scenarios. While these loading scenarios impact both the magnitude and dispersion of the calculated settlement, the overall shape of the distributions remains consistent. Moreover, the relative agreement between CPTs and Voxels models is maintained.

To allow a consistent comparison, it was required to integrate the engineering judgment in the interpretation of the Voxel realizations to better match the in-situ characteristics of the selected study area. The sand litho-classes of the GeoTOP models were herein assumed to have the same hydro-geo-mechanical properties of the superficial soil strata of the dyke (i.e., “dyke material/sand” in Fig. 4), as briefly described in Sect. 2.1. This step was required to assign the parameters for the numerical simulations (i.e., Table 1), and to allow a consistent comparison between the results of the two independent datasets removing the dependency from the material properties.

## Conclusions

In this exploratory study, the effects of the in-situ lithological heterogeneity are investigated by employing numerical simulations. The numerical models depict the behaviour of the study area at high (in-situ level, over a very fine grid ranging from 1.25 m × 5 m up to 5 m × 5 m) and low (national level, grid of 100 m × 100 m) resolutions. The effect of different loading and hydrostatic conditions is herein investigated. Thus, it is observed that:

- Settlements computed using 3D models based on the high-resolution dataset exhibit twice the relative variability, quantified by the coefficient of variation, compared to 1D analyses of the same dataset. This increased heterogeneity in the 3D model settlements highlights a more accurate representation of the varying behaviour across different parts of the study area compared to the two- and one-dimensional analyses. This suggests that 3D analyses are required to accurately account for the effects of lithological heterogeneity.
- The correlation lengths, calculated based on soil layer thickness, range from 2 to 10 m and align with those derived from the computed settlement for each corresponding soil layer. In other words, the spatial variability of the material thickness matches the spatial variability of the soil settlements due to a uniform load and excluding the variation of the material properties.
- The correlation length of the material thickness and settlement, ranging between 2 and 10 m, is in the same order of magnitude as the extent of typical structures, e.g., houses, roads and embankments. This suggests that the spatial variability of soil layer thickness can cause uneven settlement at the structural scale, potentially leading to damage.
- A good agreement is observed between the settlements computed using models based on high-resolution (in-situ level) and low-resolution (country level) datasets. Specifically, the distributions of settlements derived from the two datasets align closely in terms of their mean, dispersion, and overall shape.
- The values of the dispersion of the material thickness, quantified by the coefficient of variation, match well with those of the computed settlements for each soil layer, indicating a relationship between the two parameters, despite the CoVs referring to different metrics. For instance, for the peat layer, the CoV of the material thickness is 0.16, which closely matches the computed settlement for the peat layer the CoV ranges between 0.15 and 0.16.
- In this study, the spatial variability of the lithological variations in the soil stratigraphy, ranging between 2 and 10 m, is observed to have the same order of magnitude as that reported in previous studies for the material properties of soil layers. Therefore, the two effects could contribute with similar relevance in augmenting the differential settlements at the scale of structures.

The analyses indicate that local variation in the soil stratigraphy can be a source of unevenness in the spatial distribution of ground settlement. The findings of this study are expected to serve as a foundational step for further analyses aimed at evaluating the effects of lithological heterogeneity, combined with the inherent variability of soil properties, on buildings situated in areas susceptible to ground settlement. Additionally, this research establishes a solid basis for conducting tests or obtaining field measurements to facilitate further validation. It is hoped that this work will spark interest in the topic, inspiring future research to carry out experiments and collect field data, that could be used for cross-validation.

## Data Availability

All the sources of data used herein are reported.

## References

[CR1] Abija FA (2023) Ground variation, geotechnical uncertainties and reliability of foundation design for sustainable building infrastructures with case histories. J Mater Sci Eng Technol. 10.61440/JMSET.2023.v1.02

[CR2] Basha A, Azzam W, Elsiragy M (2024) Utilization of sand cushion for stabilization of peat layer considering dynamic response of compaction. Civ Eng J-Tehran 10(4):1182–1195. 10.28991/Cej-2024-010-04-011

[CR3] Bauduin C (2003) Uncertainties and their relevance for the design of deep excavations near existing structures. In: Geotechnical problems with man-made and man influenced ground

[CR4] Bentley (2023) Plaxis 2D - Reference Manual 2D. 2 edn

[CR5] Bezih K, Remadna AE, Remadna MS, Demagh R (2025) Reliability analysis of RC structures considering soil stiffness variability and soil-structure interaction. Geomate J 28(127):28–38. 10.21660/2025.127.4749

[CR6] Breysse D, Niandou H, Elachachi S, Houy L (2005) A generic approach to soil-structure interaction considering the effects of soil heterogeneity. Geotechnique 55(2):143–150. 10.1680/geot.55.2.143.59528

[CR7] Conte E, Dente G, Troncone A (2004) Settlements of three buildings founded on stratified soils

[CR8] Costa AL, Kok S, Korff M (2020) Systematic assessment of damage to buildings due to groundwater lowering-induced subsidence: methodology for large scale application in the Netherlands. Proc Int Assoc Hydrol Sci 382:577–582. 10.5194/piahs-382-577-2020

[CR9] de Gast T (2020) Dykes and embankments: a geostatistical analysis of soft terrain. Delft University of Technology

[CR10] de Gast T, Vardon P, Hicks M (2017) Estimating spatial correlations under man-made structures on soft soils. In Geo-Risk 2017. pp 382–389

[CR11] de Gast T, Hicks MA, Vardon P (2020) Cone penetration test (CPT) dataset to study soil heterogeneity. 4TU.ResearchData

[CR12] de Gast T, Hicks MA, Van den Eijnden AP, Vardon PJ (2021a) On the reliability assessment of a controlled dyke failure. Géotechnique 71(11):1028–1043. 10.1680/jgeot.19.SiP.003

[CR13] De Gast T, Vardon PJ, Hicks MA (2021b) Assessment of soil spatial variability for linear infrastructure using cone penetration tests. Géotechnique 71(11):999–1013. 10.1680/jgeot.19.SiP.002

[CR14] Denis A, Elachachi SM, Niandou H (2011) Effects of longitudinal variability of soil on a continuous spread footing. Eng Geol 122(3–4):179–190. 10.1016/j.enggeo.2011.05.015

[CR15] Elkateb T, Chalaturnyk R, Robertson PK (2003) An overview of soil heterogeneity: quantification and implications on geotechnical field problems. Can Geotech J 40(1):1–15. 10.1139/T02-090

[CR16] Erkens G, Kooi H, Melman R (2021) Actualisatie bodemdalingsvoorspellingskaarten. Deltares edn

[CR17] Faleih ZH, Al-Gharbawi ASA, Baqir HH (2024) The behavior of the tunnel reinforced with geogrid in soft soil under the effect of axial load. Civ Eng J-Tehran 10(8):2471–2484. 10.28991/Cej-2024-010-08-04

[CR18] Fiamingo A, Bosco M, Massimino MR (2023) The role of soil in structure response of a building damaged by the 26 December 2018 earthquake in Italy. J Rock Mech Geotech Eng 15(4):937–953. 10.1016/j.jrmge.2022.06.010

[CR19] Gibson R (1974) The analytical method in soil mechanics. Geotechnique 24(2):115–140. 10.1680/geot.1974.24.2.115

[CR20] Han J, Huang J, Parsons RL (2007) Influence of bedrock inclination on elastic settlements of flexible shallow strip foundations. Comput Geotech 34(1):53–56. 10.1016/j.compgeo.2006.09.004

[CR21] Houy L, Breysse D, Denis A (2005) Influence of soil heterogeneity on load redistribution and settlement of a hyperstatic three-support frame. Geotechnique 55(2):163–170. 10.1680/geot.55.2.163.59524

[CR22] Ibrahim M, Al-Obaydi M (2021) Numerical analysis of a rectangular footing resting on two inclined layers of soil. In: IOP conference series: earth and environmental science. IOP Publishing, p 012039

[CR23] Jamshidi Chenari R, Pourvahedi Roshandeh S, Payan M (2019) Stochastic analysis of foundation immediate settlement on heterogeneous spatially random soil considering mechanical anisotropy. SN Appl Sci 1:1–15. 10.1007/s42452-019-0684-0

[CR24] Koster K, Stafleu J, Stouthamer E (2018) Differential subsidence in the urbanised coastal-deltaic plain of the Netherlands. Neth J Geosci 97(4):215–227. 10.1017/njg.2018.11

[CR25] Kruiver PP, Wiersma A, Kloosterman FH, de Lange G, Korff M, Stafleu J et al (2017) Characterisation of the Groningen subsurface for seismic hazard and risk modelling. Neth J Geosci-Geologie En Mijnbouw 96(5):S215–S233. 10.1017/njg.2017.11

[CR26] Krzysztof N (2019) Numerical analysis of settlement of a structure situated on a heterogeneous loess subsoil. In: IOP conference series: materials science and engineering. IOP Publishing, p 052036

[CR27] Kumar V, Burman A, Portelinha F, Kumar M, Das G (2024) Influence of variation of soil properties in bearing capacity and settlement analysis of a strip footing using random finite element method. Civ Eng Infrastruct J 57(2):383–403. 10.22059/ceij.2023.360871.1930

[CR28] NEN9997–1+C2:2017nl (2017) Geotechnisch ontwerp van constructies - Deel 1: Algemene regels (Geotechnical design of structures - Part 1: General rules)

[CR29] Peduto D, Nicodemo G, Maccabiani J, Ferlisi S (2017) Multi-scale analysis of settlement-induced building damage using damage surveys and DInSAR data: a case study in The Netherlands. Eng Geol 218:117–133. 10.1016/j.enggeo.2016.12.018

[CR30] Peduto D, Prosperi A, Nicodemo G, Korff M (2022) District-scale numerical analysis of settlements related to groundwater lowering in variable soil conditions. Can Geotech J 99(999):1–16. 10.1139/cgj-2021-0041

[CR31] Popescu R, Deodatis G, Nobahar A (2005) Effects of random heterogeneity of soil properties on bearing capacity. Probab Eng Mech 20(4):324–341. 10.1016/j.probengmech.2005.06.003

[CR32] Prosperi A, Nicodemo G, Korff M, Peduto D (2022) Multi-source monitoring data and numerical analyses for the assessment of settlements affecting built-up areas in variable soil conditions. In: 11th international symposium on field monitoring in geomechanics (ISFMG2022) ISSMGE)

[CR33] Stafleu J, Maljers D, Gunnink JL, Menkovic A, Busschers FS (2011) 3D modelling of the shallow subsurface of Zeeland, the Netherlands. Neth J Geosci-Geologie En Mijnbouw 90(4):293–310. 10.1017/S0016774600000597

[CR34] Stafleu J, van der Meulen MJ, Gunnink JL, Maljers D, Hummelman J, Busschers FS et al. (2019) Systematic 3D subsurface mapping in the Netherlands. In Synopsis of current three-dimensional geological mapping and modelling in geological survey organizations. Alberta Energy Regulator/Alberta Geological Survey, AER/AGS Special Report, vol 112, pp 179–190

[CR35] Stafleu J, Maljers D, Busschers FS, Schokker J, Gunnink JL, Dambrink RM (2021) Models created as 3‐D cellular voxel arrays. In: Applied multidimensional geological modeling: informing sustainable human interactions with the shallow subsurface. 10.1002/9781119163091.ch11

[CR36] Uzielli M, Lacasse S, Nadim F, Phoon KK (2006) Soil variability analysis for geotechnical practice. Charact Eng Prop Nat Soils 3:1653–1752. 10.1201/NOE0415426916.ch3

[CR37] Vanmarcke E (1984) Random Fields: Analysis and Synthesis, Published by MIT Press, Cambridge MA, 1983; Web Edition by Rare Book Services, Princeton University, Princeton NJ, 1998

[CR38] Verberne M, Koster K, Fokker PA (2024) Multi-data settlement prediction along a road section integrating InSAR and coastal subsurface information with data assimilation. Front Earth Sci 11:1323874. 10.3389/feart.2023.1323874

[CR39] Viviescas JC, Griffiths D, Osorio JP (2022) Geological influence on the spatial variability of soils. Int J Geotech Eng 16(3):382–390. 10.1080/19386362.2021.1888509

[CR40] Yuan WH, Wang HC, Li YJ, Zhang W, Liu K (2024) Large deformation assessment of the bearing capacity factor for rigid footing: effect of soil heterogeneity. Comput Part Mech 11(6):2923–2941. 10.1007/s40571-024-00763-6

[CR41] Zhang S, Wang Y, Gao Q, Ma X, Zhou H, Wang Z (2024) Probabilistic analysis of ground settlement induced by tunnel excavation in multilayered soil considering spatial variability. Comput Geotech 165:105951. 10.1016/j.compgeo.2023.105951

